# Audiological Effects of COVID-19 Infection: Results of a Standardized Interview

**DOI:** 10.1017/cjn.2021.179

**Published:** 2021-07-21

**Authors:** Veronika Vielsmeier, Steven C. Marcrum, Franziska C. Weber, Berthold Langguth, Constantin Hintschich

**Affiliations:** University of Regensburg, Department of Otorhinolaryngology, Regensburg, Germany; University of Regensburg, Interdisciplinary Tinnitus Centre, Regensburg, Germany; University of Regensburg, Department of Psychiatry and Psychotherapy, Regensburg, Germany

**Keywords:** Tinnitus, hearing loss, dizziness, COVID-19

It was with great interest which we read the recent article “Hearing Loss, Tinnitus, and Dizziness in COVID-19: A Systematic Review and Meta-Analysis” by Jafari et al.^1^ Since SARS-CoV-2 infections first began overwhelming medical infrastructures in early 2020, more than 175 million cases of the novel coronavirus have been confirmed, with the number growing in excess of 500,000 cases per day (WHO COVID-19 dashboard, 2021, June 9; retrieved from https://covid19.who.int/). Over the past 18 months, enormous progress has been made in identifying a wide array of clinical manifestations of COVID-19 infection, spanning the range from olfactory and gustatory deficits to impaired consciousness and encephalopathy.^2–4^ However, despite a rapidly growing literature base, relatively limited data are available concerning involvement of the auditory system. This is especially unfortunate given that viral infections have long been known to cause hearing loss, tinnitus, and other audio-vestibular symptoms.^4–6^ With this letter, we would like to report findings from our standardized interview-based evaluation of hearing impairment, tinnitus, and hyperacusis in a sample of adult COVID-19 patients. In so doing, we hope to contribute meaningfully to the understanding of a significant, yet under-evaluated topic.

In our study, 28 adults with SARS-CoV-2 infection, as verified via real-time reverse transcription polymerase chain reaction (PCR), were interviewed 4 to 5 months post-infection (mean = 140 d; SD = 14 d) regarding audiological symptoms experienced during the period of acute infection. As the goal of this study was to evaluate for strong effects of COVID-19 infection on the auditory system, such as which could efficiently orient future research, inclusion criteria required only that a participant 1) be 18 years of age at the time of acute infection and 2) have had a positive PCR test for COVID-19. These criteria provided the benefit of not excluding participants with symptoms insufficient to require hospitalization, thereby making the results more representative of the course for the typical person infected with COVID-19.

The standardized interview was designed to explore the areas of hyperacusis, hearing impairment, and tinnitus. Briefly, questions were posed, such as (1) During your infection with the Coronavirus, did you experience sounds or noises in your everyday life as being louder or more uncomfortable than usual?; (2) During your infection with the Coronavirus, was your ability to hear different than usual?; or (3) During your infection with the Coronavirus, did you experience tinnitus, or noises in your ears or head, for longer than five minutes? Possible responses to all questions were either ‘no’, ‘yes – new complaint’, or ‘yes – worsening of existing complaint’.

Twenty-eight adults (18 females, 10 males) with a confirmed history of COVID-19 infection were included in this study. Overall, 14.3% (four persons) of participants reported some type of audiological symptom during their interview (see Table 1). Briefly, 10.7% (three persons) reported experiencing tinnitus during their SARS-CoV-2 infection, while 7.2% (two persons) reported either a worsening of hearing ability or an increased sensitivity to sound. Of the four persons reporting audiological symptoms, one reported both hearing loss and tinnitus, whereas the other three reported a single audiological symptom. Of clinical importance, two participants reported that audiological symptoms were still present at the time of the interview, while the other two participants reported symptoms having resolved prior to the interview. Finally, no clear relationship between the severity of general COVID-19 symptoms and the propensity for audiological involvement could be identified; however, as none of the included participants required hospitalization, the restricted range of disease courses observed in this study might have served to obscure a meaningful relationship.

**Table 1:** Results of our patients

	No	New complaints	Worsening
Tinnitus	89.3% (25)	10.7% (3)	0% (0)
Hearing impairment	96.4% (27)	3.6% (1)	0% (0)
Hyperacusis	96.4% (27)	3.6% (1)	0% (0)

The primary aim of this study was to evaluate a sample of adults for audiological symptoms associated with acute COVID-19 infection. Results indicated auditory system involvement in a meaningful proportion of participants (14.3%), suggesting that additional work investigating the potential relationship between COVID-19 infection and audiological outcomes is merited. Further, results of the present study suggest that, in contrast to the majority of previous works which focused largely on hospitalized patients or those with severe symptoms, inclusion criteria encompassing patients with less severe symptoms should also be considered.

Recent epidemiological studies report an incidence of tinnitus in the general, adult population of approximately 25 new tinnitus cases per 10,000 person-years.^7^ Based on this figure and assuming a typical duration of COVID-19 infection of 3 weeks, it would be reasonable to expect 0.004 tinnitus cases in our sample (28 patients × 3 weeks/patient). However, the incidence of tinnitus observed in this study greatly exceeded this expectation. While this finding by no means establishes a causal relationship between COVID-19 infection and auditory system dysfunction, it is consistent with the existence of such a relationship and underlines the need for future investigations.

It is plausible, for example, that the increased risk of tinnitus onset in acute COVID-19 infection is not directly related to the viral infection itself, but rather is mediated by associated, non-specific factors, such as stress. However, several hypotheses have been reported, which describe mechanisms by which COVID-19 infection might deleteriously alter the auditory system. Among them are (i) direct infection of the brain or cochlea, as a postmortem case series including 43 patients reported SARS-CoV-2 viral proteins in brain tissue,^8^ (ii) the result of vasculopathy or an inflammatory reaction due to SARS-CoV-2 infection,^3,8^ or even (iii) side effects of COVID-19 treatments, such as chloroquine. However, to date, it is not possible to definitively state the existence of a causal relationship between COVID-19 infection and auditory system dysfunction.

The generalizability of this study’s results is limited by several factors, including the number of included participants, the lack of a control group, and the lack of longitudinal findings. However, the results nonetheless suggest that neuro-otological symptoms, such as tinnitus, hearing impairment, and hyperacusis, may affect a significant proportion of adults during acute COVID-19 infection, even if those patients experience an otherwise mild disease course. Further studies are warranted to better elucidate effects of COVID-19 on the auditory system, as well as to better identify underlying pathophysiological mechanisms.
